# Internal Carotid Artery Agenesis: A Rare Entity

**DOI:** 10.7759/cureus.36640

**Published:** 2023-03-24

**Authors:** Ashish Kulhari, Amrinder Singh, Farah Fourcand, Haralabos Zacharatos, Siddhart Mehta, Jawad F Kirmani

**Affiliations:** 1 Department of Neurology, Research Medical Center, Kansas City, USA; 2 Department of Neurology, University of Missouri Kansas City School of Medicine, Kansas City, USA; 3 Department of Medicine, Kansas City University of Medicine and Biosciences, Kansas City, USA; 4 Department of Neurology, United Health Services (UHS) Binghamton General Hospital, Johnson City, USA; 5 Department of Neurology, Hackensack Meridian Health John F. Kennedy (JFK) Medical Center, Edison, USA

**Keywords:** carotid agenesis, stroke, aneurysm, stroke intervention, stent-assisted coil

## Abstract

Congenital absence of the internal carotid artery (ICA) is an extremely rare entity that occurs due to insult during the embryonic development of the ICA. Various intracranial collateral pathways develop to compensate for the ICA agenesis. Patients can present with aneurysmal subarachnoid hemorrhage, stroke-like symptoms, or other neurological symptoms due to compression of brain structures from enlarged collateral pathways/aneurysms. We present two cases of ICA agenesis along with an extensive review of the literature.

A 67-year-old man presented with fluctuating right-sided hemiparesis and aphasia, found to have left ICA agenesis. The left middle cerebral artery (MCA) is supplied by the basilar artery through the well-developed posterior communicating artery (PCOM). Left ophthalmic artery coming from the proximal left MCA.

A 44-year-old woman presented with severe headaches, found to have right ICA agenesis with bilateral MCAs and anterior cerebral arteries (ACA) supplied by left ICA. A 17-mm anterior communicating artery (ACOM) aneurysm was discovered.

## Introduction

Congenital absence of the internal carotid artery (ICA) is extremely rare with an incidence estimated to be <0.01% [1]. ICA agenesis occurs due to insult during the embryonic development of the ICA. Intracranial collateral pathways form to compensate for the ICA agenesis. The development of collateral pathways is dependent on the timing of the embryonic insult. Primitive collateral pathways like inter-cavernous anastomosis dominate if embryonic insult occurs before the development of the circle of Willis and the circle of Willis collaterals dominates if insult occurs after the development of the circle of Willis. The absence of a carotid canal on the computed tomography (CT) head along with the absence of ICA on digital subtraction angiography (DSA) confirms the diagnosis of ICA agenesis. Given the extremely rare entity, differentiating ICA agenesis from atherosclerotic steno-occlusive and dissection is very challenging especially when the patient presents with stroke-like symptoms. There are very few cases of ICA agenesis in literature. Through this manuscript, we report our two cases of ICA agenesis along with an extensive review of the literature.

## Case presentation

Case 1

**Figure 1:**
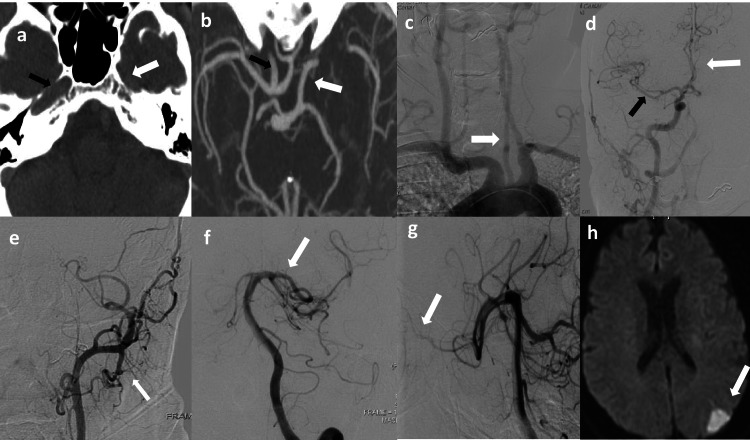
(a) CT head shows the absence of the left carotid canal (white arrow) and the well-formed right carotid canal (black arrow). (b) CT angiogram head shows basilar artery supplying left MCA through well-developed PCOM (white arrow) and right ICA supplying bilateral ACA and ipsilateral MCA (black arrow). (c) Aortic arch injection reveals a smaller caliber of the left common carotid artery (white arrow). (d) Right common carotid injection (AP view) shows right ICA suppling bilateral ACA (white arrow) and ipsilateral MCA (black arrow). (e) Left common carotid injection (AP view) reveals the absence of left ICA and opacification of ECA branches (white arrow). (f) Left vertebral artery injection (AP view) showing the well-developed PCOM (white arrow) supplying left MCA. (g) Left vertebral artery injection (Lateral view) shows the ophthalmic artery (white arrow) coming off the MCA. (h) MRI brain showing left MCA-PCA acute watershed infarct (white arrow). CT: computed tomography; MCA: middle cerebral artery; PCOM: posterior communicating artery; ICA: internal carotid artery; ACA: anterior cerebral artery; ECA: external carotid artery; MRI: magnetic resonance imaging; AP: anterior-posterior

A 67-year-old right-handed Hispanic man with a past medical history of hypertension, cigarette smoking, and coronary artery disease was brought to our emergency room (ER) for sudden onset of fluctuating right-sided hemiparesis, dysarthria, and global aphasia. On arrival at the ER, the patient was almost back to normal (NIHSS 0). CT head did not show any hemorrhage, hyperdense sign, or loss of gray-white differentiation but did reveal the absence of a left carotid canal (Figure 1a). CT angiogram of the head showed the absence of left ICA, the basilar artery supplying the left middle cerebral artery (MCA) through the prominent left posterior communicating artery (PCOM), and the right ICA supplying both the anterior cerebral arteries (ACA) (Figure 1b). MRI brain showed a left middle cerebral artery-posterior cerebral artery (PCA) watershed infarct (Figure 1h). The patient was started dual antiplatelet therapy with permissive hypertension along with fluid resuscitation. The next day morning, the patient had sudden onset of similar symptoms. The patient was not a candidate for IV thrombolytics due to a recent cerebral infarct. The patient was immediately taken to a neuro-interventional suite for endovascular therapy. DSA showed a smaller caliber of the left common carotid artery (Figure 1c), right ICA supplying bilateral ACA’s and ipsilateral MCA (Figure 1d), absence of left ICA (Figure 1e) with basilar artery supplying left MCA territory through well-developed PCOM (Figure 1f) and left ophthalmic artery originating from proximal left MCA (Figure 1g). No large vessel occlusion was found. Intra-arterial antiplatelet agent (Eptifibatide) and vasodilator (Verapamil) were infused to improve the microcirculatory flow in the area of hypoperfusion. The patient's neurological symptoms improved post-endovascular therapy.

Case 2

**Figure 2:**
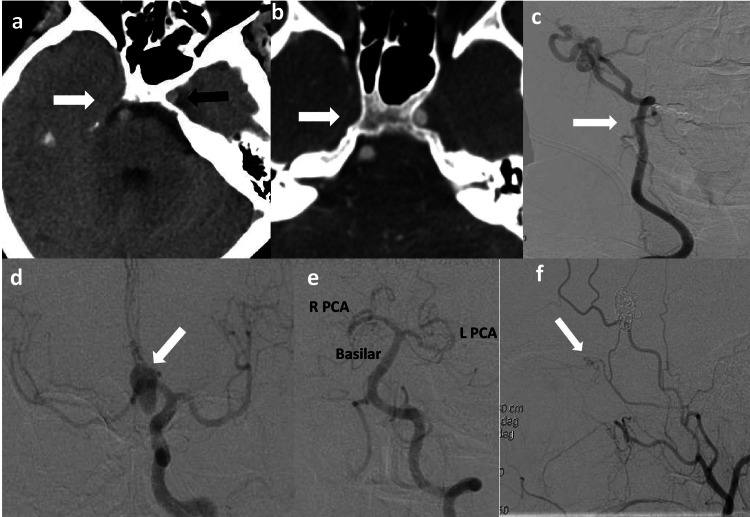
(a) CT head shows the absence of the right carotid canal (white arrow) and the well-formed left carotid canal (black arrow). (b) CT angiogram head shows the absence of right ICA (white arrow). (c) Right common carotid injection (AP view) reveals the absence of right ICA and opacification of ECA (white arrow). (d) Left common carotid injection (AP view) shows the large ACOM aneurysm (white arrow) with bilateral MCA’s and ACA’s being supplied by Left ICA. (e) Left vertebral artery injection (AP view) shows basilar and bilateral posterior cerebral arteries (PCA), but did not reveal any collaterals from posterior to anterior circulation. (f) Left common carotid injection (Lateral view) shows an ophthalmic artery (white arrow) coming off the MMA. CT: computed tomography; ICA: internal carotid artery; AP: anterior-posterior; ECA: external carotid artery; ACOM: anterior communicating artery; MCA: middle cerebral artery; ACA: anterior cerebral artery; PCA: posterior cerebral artery; MMA: middle meningeal artery

A 44-year-old right-handed Hispanic woman with a history of hypertension, cigarette smoking, and no family history of aneurysms, presented to ER for severe, persistent, bifrontal headaches. CT head did not show any hemorrhage but was read as suspicious of a large anterior communicating artery aneurysm. CT head also showed an absence of the right carotid canal (Figure 2a). CT angiogram head showed an absence of right ICA (Figure 2b). DSA showed a small caliber of right CCA, absence of right ICA (Figure 2c), left ICA supplying bilateral ACAs and MCAs, 17 mm anterior communicating artery (ACOM) aneurysm (Figure 2d), right ophthalmic artery originating from a frontal branch of the right middle meningeal artery (MMA) (Figure 2f). The patient underwent successful stent-assisted coil embolization of the aneurysm.

## Discussion

Background

Tode accidentally discovered the first case of carotid agenesis during a post-mortem examination in 1787 [2]. Verbiest reported the first case of carotid agenesis by cerebral angiography in 1954 [3]. In 1968, Lie defined agenesis as a complete failure of an organ to develop, aplasia as the lack of development of an organ, and hypoplasia as the incomplete development of an organ [4]. Although the exact etiology of ICA developmental anomalies is unknown, all three variations are thought to represent the sequela from an insult to the developing embryo [5].

Embryology

Consensus exists on the origin of ICA from the third aortic arch but the jury is still out on whether the origin of the external carotid artery (ECA) occurs jointly with the proximal ICA or independently from the aortic arch. Given most of the cases of ICA agenesis have normal ECA, the theory of independent development of ICA and ECA seems more plausible [3,4,6,7]. Origin of ICA from the dorsal aorta and the third aortic arch starts at approximately 4- to 5-millimeters (mm) embryonic stage, with the full development of ICA by six weeks [7,8]. Embryonic development of ICA is a very complex process involving multiple simultaneous steps. Any disruption in any of these steps can lead to developmental anomalies of ICA. Carotid atresia, absence of sympathetic plexus, anomalous origin of ophthalmic artery from the MCA or middle meningeal artery, and other associated anomalies are associated with ICA agenesis [9].

Lie classified the collateral pathways associated with ICA agenesis into six categories (Types A-F) (Table 1) [6]. Lie’s original six collateral pathways can be simplified into three main types: collateral flow through the circle of Willis (most frequent), collateral flow via persistent fetal circulation, and reconstitution of the ICA through skull base collaterals from the ECA [10,11]. Development of these collateral pathways is dependent on the timing of the embryonic insult, before or after the development of the circle of Willis. Primitive collateral pathways like inter-cavernous anastomosis dominate if embryonic insult occurs before the development of the circle of Willis (24-mm embryonic stage). Likewise, collateral flow through the circle of Willis dominates if insult occurs after the development of the circle of Willis [12].

**Table 1:** Lie’s classification of collateral pathways in ICA agenesis [6]. ICA: internal carotid artery; ACA: anterior cerebral artery; ACOM: anterior communicating artery; MCA: middle cerebral artery; PCOM: posterior communicating artery; ECA: external carotid artery

Lie type	Collateral pathways in ICA agenesis
A	In presence of unilateral ICA agenesis, collateral circulation to the ipsilateral ACA through a patent ACOM and to the ipsilateral MCA from the posterior circulation through a hypertrophied PCOM.
B	In presence of unilateral ICA agenesis, collateral circulation to the ipsilateral ACA and MCA through a patent ACOM.
C	In presence of bilateral ICA agenesis, the entire anterior circulation is supplied through carotid-vertebrobasilar anastomoses via hypertrophied PCOM.
D	In presence of unilateral ICA agenesis, intercavernous communication to the ipsilateral carotid siphon from the contralateral cavernous ICA.
E	Diminutive ACAs are supplied by bilateral hypoplastic ICAs or in presence of unilateral ICA agenesis from contralateral hypoplastic ICA, and the MCAs are supplied by hypertrophied PCOMs.
F	Collateral flow to the ICA through transcranial anastomoses from the internal maxillary branches of the ECA system.

Clinical features

As per a recently published large retrospective study, clinical features can be divided according to the age groups (0-20 years, 20-40 years, and >40 years) [9]. Developmental delay (54%) and subarachnoid hemorrhage (SAH) like symptoms (25%) including headache, nausea, and vomiting, were the most common symptoms in the 0- to 20-year-old group. Developmental delay is often attributed to abnormal development of the pituitary or blood supply to the pituitary. SAH symptoms are attributed to aneurysm formation and rupture due to alteration of intracranial hemodynamics [9]. The estimated prevalence of cerebral aneurysms in the general population is 2% to 4%, but the reported prevalence of aneurysms in association with the absence of the ICA is 24% to 34% [4,7,9,13]. Almost half of the patients in the age group older than 40 years presented with transient ischemic attacks and ischemic stroke. This could be attributed to the decompensation of the collateral circulation due to atherosclerosis [9]. Although patients in the 20- to 40-year-old group can present with any of the above symptoms, most of the asymptomatic cases were noted in this group (12.5%). This could be explained by the adaptation of the brain to the intracranial collateral pathways [9]. Some other symptoms that can be attributed to compression from enlarged collateral pathways are visual field defects, ocular palsy, trigeminal neuralgia, pulsatile tinnitus, etc. [7,9].

Radiographic findings

Embryonic development of ICA precedes the development of the base of the skull. The presence of ICA or its precursor is a prerequisite for the development of the carotid canal [7,9]. Therefore, the presence or absence of a carotid canal on the CT head is the key to differentiating aplasia from agenesis. The absence of a carotid canal on the CT head along with the absence of ICA on angiography will confirm the diagnosis of ICA agenesis.

Anomalous origin of the ophthalmic artery is noted in ICA agenesis. The ophthalmic artery can originate from the ipsilateral middle meningeal artery or rarely from the proximal portion of the MCA (as reported in our cases) [9].

Having knowledge of collateral pathways and ophthalmic artery origin in ICA agenesis is very important, especially during transarterial embolizations, trans-sphenoidal hypophyseal surgery, carotid endarterectomy, etc. [7,9].

Differential diagnosis

Because of the extreme rarity of the condition and non-specific clinical features, diagnosing ICA agenesis/hypoplasia can be very challenging especially if the patient is presenting with stroke-like symptoms. Common ICA pathologies like atherosclerotic steno-occlusive disease and dissections should be considered in the differential diagnosis. The absence of a carotid canal on the CT head as discussed above is the hallmark feature of ICA agenesis. Other key radiographic signs including the presence of calcifications at the ICA origin in case of atherosclerotic steno-occlusive disease and the presence of blood flow in the proximal extracranial ICA in most of the cases of dissection can be very helpful for differentiating them from ICA agenesis.

Treatment

Effective therapy for congenital absence of the ICA is currently unavailable. Symptomatic treatment and collateral circulation pathway reconstruction, like carotid stenting or carotid endarterectomy in case of extracranial ICA stenosis, are the main treatment methods to maintain compensative arterial blood flow and reduce blood flow resistance. Dolichoectasia of collateral pathways and large aneurysms can cause neurological symptoms due to the compression of normal brain structures. Aneurysm treatment helps alleviate pressure on the normal brain structures [9].

## Conclusions

Congenital absence of the ICA is an extremely rare entity that occurs due to insult during the embryonic development of the ICA. We present two cases of ICA agenesis along with an extensive review of the literature. Given the rarity of this condition, we believe our cases will robust the literature on ICA agenesis.
